# The Role of an Online Community for People With a Rare Disease: Content Analysis of Messages Posted on a Primary Biliary Cirrhosis Mailinglist

**DOI:** 10.2196/jmir.7.1.e10

**Published:** 2005-03-31

**Authors:** Judith N Lasker, Ellen D Sogolow, Rebecca R Sharim

**Affiliations:** ^1^Department of Sociology and AnthropologyLehigh UniversityBethlehem, PAUSA

**Keywords:** Internet support, chronic liver disease, primary biliary cirrhosis

## Abstract

**Background:**

This study focuses on the role of an Internet-based group for people who have an autoimmune liver disease, primary biliary cirrhosis. Primary biliary cirrhosis is a relatively rare disease, affects primarily women in their 40's and older, and is not well understood. The PBCers Organization (PBC stands for primary biliary cirrhosis) provides electronic mailinglists (listservs) and informational resources for those with primary biliary cirrhosis.

**Objectives:**

(1) to identify the issues of greatest importance to those posting to the listserv, specifically the relative importance of biomedical, socioemotional, and organizational/systems messages; (2) to compare frequency and content of posts by people at different stages of disease; (3) to identify how people with primary biliary cirrhosis represent the psychosocial challenges and dilemmas (role and identity change, uncertainty, and stigma) identified in the social-scientific literature as key elements of the experience of chronic disease.

**Methods:**

The paper is based on content analysis of messages posted during two months to the Daily Digest listserv for people who have primary biliary cirrhosis. To analyze the posts, we developed a coding system with three major categories--biomedical, socioemotional, and systems/organizations--and 12 codes in each category.

**Results:**

A total of 275 people posted 710 messages. Of the 250 people for whom information on gender was available, 239 (95.6%) were women and 11 (4.4%) were men. Analysis of 710 messages posted to the listserv revealed a predominance of requests for and reports of biomedical information, such as health care providers (32.7%), medications (30.9%), tests and procedures (25.8%), and symptoms (25.7%), combined with very frequent expressions of emotional support. The most frequent single topics were peer support (included in 40.6% of all posts) and positive emotions (25.3%). Posters who reported fewer years since diagnosis were more likely to be seeking biomedical information than those who were further in time from their diagnosis (r= -.241, P<.001, n=313). Those in later stages posted an average of 3.87 messages, compared to an average of 2.64 for people in earlier stages (t= 1.786, P=.08, n=90), which is different from what we expected. No relation between years since diagnosis or age and number of messages was found. Contrary to our expectations, the topics reflecting issues of role change/identity (2.9%), stigma (0.7%), and thoughts about the future (3.9%), all identified in social-scientific literature as key concerns for people with chronic illness, appeared infrequently in this set of messages.

**Conclusions:**

Messages exchanged on this particular mailing list have a biomedical, rather than socioemotional or organizational, emphasis. The Internet offers a highly valued opportunity for those with rare diseases to connect with, learn from, and provide support to others having similar experiences. Research that compares those with primary biliary cirrhosis, who are involved in an Internet support group and those who are not, would be an important next step to better understanding the role of the Internet among patients with chronic liver disease and the implications of it in the course of their illness.

## Introduction

### Primary Biliary Cirrhosis

Primary biliary cirrhosis is not a well-known disease, yet it is emblematic of two growing phenomena in modern medicine—the increasing prevalence of autoimmune disorders among women, and the increasing demand for and survival following organ transplantation. Primary biliary cirrhosis is one of the autoimmune disorders that areestimated to affect between 14 and 22 million Americans. Nearly all these disorders affect women at several times the rate at which they affect men; 90% of people with primary biliary cirrhosis are women. In fact, autoimmune diseases rank in the top ten causes of death of American women in every age group under 65. A 2002 report of the US National Institutes of Health (NIH) asserts that “autoimmune diseases . . . represent a significant physical, emotional, social, and fiscal burden to the country's health care system.” [1] Just as importantly, they represent significant physical, emotional, social, and fiscal challenges to the families of these patients. Yet they are often poorly understood, misdiagnosed, and constructed as psychological, ie, not ”real”, with accompanying stigma and lack of validation of the women's physical and psychological experiences. Primary biliary cirrhosis is further stigmatized due to the assumption that any liver disease must be caused by substance abuse.

In the last ten years, over 30000 women have received liver transplants in the United States, and primary biliary cirrhosis is the second leading diagnosis in this group. Current data shows that liver disease is the seventh leading cause of death in the United States among Americans 25 to 64 years of age [2]. Primary biliary cirrhosis is one type of liver disease that is often debilitating and may be fatal. With primary biliary cirrhosis, the body attacks the cells lining the liver's bile ducts, causing inflammation and destruction. In advanced stages, it may lead to the possibility of a liver transplant for those who have adequate resources. The most common symptoms of primary biliary cirrhosis are debilitating fatigue and itching, affecting respectively, 65% to 85% and 25% to 70% of people with primary biliary cirrhosis. People with primary biliary cirrhosis usually also suffer from hyperlipidemia and osteoporosis. Potentially life-threatening symptoms that may occur in advanced stages of the disease are encephalopathy (inflammation of the brain, causing confusion and cognitive dysfunction), ascites (fluid in the abdomen that can become infected) and varices (swollen vessels in the esophagus that can rupture) (JNL, EDS and LM Short, unpublished data, 2005) [3].

Globally, an estimatedfive out of 100000 people have primary biliary cirrhosis [4]. Using this rate, one can estimate that primary biliary cirrhosis probably affects around 15000 people in the United States. The National Institute of Health's Office of Rare Diseases includes primary biliary cirrhosis in its list of 6000 diseases currently recognized as rare because they affect fewer than 200000 people in the United States.

With primary biliary cirrhosis, as with many other health concerns, women seek to empower each other to cope better with the challenges they face. Many women have organized to share information and support with each other and have become activists demanding greater funding and access to care [5]. The Internet is one major tool in the transformation of the experience of illness that is taking place, as well as offering a major source of information and support for people with health concerns [6].

A study of primary biliary cirrhosis offers an opportunity to focus on how women with a rare disease use the Internet and what issues are of greatest concern to them. Our purpose is to gain a better understanding of the needs and concerns of people with primary biliary cirrhosis who are participating in a computer-mediated support group. This paper is based on the results of a content analysis of 2 months of messages posted in 2003 to a listserv for people with primary biliary cirrhosis.

### The Value of the Internet for People With Chronic Disease

As computers become more accessible to the general population, health-related searches have become one of the top 3 most common reasons for using the Internet [7]. About 4.5% of all searches on the Web are health-related [8], and it is estimated that as many as 93 million Americans have utilized the Internet for health related information [9].

One of the leading sources of health information online is from support communities that link people who have common problems with each other; a major reason for the growth of these networks is convenience of access [9]. Studies of people using such virtual networks [10-12] report the advantages of 24-hour availability of information and support from others who may be far away. Even with strong networks of support from family and friends, patients maybenefit from having an outlet of people who can relate to what they are going through on a personal level. Traditional face-to-face support groups can offer this support, but issues such as transportation, distance, privacy, and time restrictions typicallyreduce participation and attendance [12]. Thus, online support groups provide a particularly valuable alternative for people suffering from a relatively rare disease. It is often difficult for them to find medical specialists near their local communities; it is also more difficult for them to find others with the same or similar conditions with whom to share their experiences.

Two major reasons that people use the Internet for health-related concerns repeatedly emerge from reviews: first,to find biomedical information, and second, to interact with others who have similar conditions for the purpose of sharing experiences and emotional support. Online groups generally provide some combination of both information and emotional support [10]. White and Dorman [13] concluded from their study of an Internet mailgroup for caregivers of people with Alzheimer's disease that the leading type of message posted by users involved seeking or giving information. In contrast, in Finn's [14] study of an online group for disabled individuals, the majority of messages were coded as being primarily socioemotional in orientation, such as expressing feelings and providing support and empathy, rather than biomedical (task-oriented).

Klemm et al [15] consider that differences in the relative frequency of biomedical vs socioemotional issues may be related to gender differences in communication about health online. In their study of posts to 3 online cancer support groups (one for breast cancer, one for prostate cancer, and one mixed-sex group for cancer in general), they found that women were more likely to communicate support and encouragement, while men were more likely to communicate information. It may be, however, that in the case of a rare disease that is poorly understood and misdiagnosed, the need for specific biomedical information would take precedence.

Prior studies [9] indicate that younger and/or more educated people are most likely to rely on the Internet for health information; thus characteristics other than gender are potentially important predictors of Internet use in the case of a support network for chronic disease.

### Psychosocial Challenges of Chronic Illness

Social scientists who study the experience of chronic illness have identified psychological and social challenges that are caused by such illness. In particular, they focus on 3 issues: (1) the need to create a new identity, a new sense of self that corresponds to the illness experience, including changes in social roles related to family, work, and social relationships; (2) the need to manage stigma related to the illness itself and to the limitations it creates; and (3) a pervasive sense of uncertainty with regard to the future [16-18]. Charmaz [18] suggests that, as a result of these challenges, chronic illness poses the major problems of making sense of bewildering symptoms, reconstructing order, and maintaining control over life.

Very few studies focus on the psychosocial consequences of chronic liver disease specifically. An exception is Wainwright's [19] interviews of 10 posttransplant patients about their lives prior to transplant. He identified 4 key concerns: uncertainty arising from becoming ill; the desire to maintain independence despite debilitating symptoms; acceptance of oneself as disabled once symptoms became more severe; and the feeling of being judged by others as alcoholic, regardless of disease etiology. These are consistent with the emphasis on identity and role change, stigma, and uncertainty found in the literature on chronic illness in general.

Liver disease differs from other chronic diseases in several important ways. First, there are major symptoms such as fatigue, ascites (fluid in abdomen), pruritus (itching), and encephalopathy (brain dysfunction) that do not have the same prominence in other diseases. Consequently these symptoms are not well understood by others. We know, for example, that diseases that have fatigue as a major symptom (particularly if they mostly affect women, the case with primary biliary cirrhosis) are often discounted as psychiatric in etiology [20]. Second, liver disease is frequently stigmatized due to its association with substance abuse. There may be stigma associated with inability to function normally and a presumption of hypochondria when the person has no visible signs of the disease.

Additionally, when liver disease is progressive, it may be fatal except for the possibility of a liver transplant. The lack of recognition for chronic liver disease symptoms, the unpredictability of chronic liver disease prognosis, the scarce resources, uncertainty, and long waits associated with transplantation combine to present unique challenges for people with liver diseases with regard to negotiating role changes, reconstructing identity, and managing uncertainty and stigma. The experience of people with primary biliary cirrhosis gives us a unique opportunity for insight into this range of issues.

### The PBCers Organization

The PBCers Organization is the largest and only US-based Internet support website for people with primary biliary cirrhosis. It provides informational and emotional support for people with primary biliary cirrhosis as well as fund-raising, advocacy, and educational programs. The organization provides services to people with primary biliary cirrhosis and to their family members and friends through a variety of mechanisms, including listservs, chatrooms, message boards, and other informational resources. An online daily digest, compiled by a team of moderators, is mailed Monday through Friday and some weekends. The PBCers Organization also offers separate listservs for specialized groups or interests: Family and Friends, Spiritual Side, Weight Loss, and Post-Transplant. The organization has local chapters and convenes conferences at which it hosts medical experts and raises money for research.

The PBCers Organization offers an opportunity to examine how people with one type of rare chronic disease utilize the Internet to enhance their health and quality of life. The organization was established in 1996 by a few people with primary biliary cirrhosis who lived far apart and began corresponding with each other by email. There are currently over 2400 members (persons with primary biliary cirrhosis, family members, friends, and health professionals) worldwide [21].

### Hypotheses

At the outset, we raised 3 questions. The following hypotheses emerged from these questions:

1. Why do people with primary biliary cirrhosis turn to an Internet-based support group? Is it primarily for medical information that will help them manage their disease, or is it mostly for emotional support from peers? There is very little research on this question. The study by Klemm et al [15] could lead us to expect that because primary biliary cirrhosis is a disease primarily affecting women, the messages would be dominated by *socioemotional* expressions of support. On the other hand, due to having a rare disease, we might expect people with primary biliary cirrhosis to be seeking *biomedical* information that most nonspecialist physicians would be unfamiliar with and that is not easily accessible otherwise. While there are reasons to support either possibility, our preliminary research on the organization led us to hypothesize that there would be greater attention to biomedical information. We based this in part on the PBCers Organization's emphasis on education, and their practice of establishing separate lists for conversations about spiritual and political issues. For instance, in a separate study of the PBCers Organization listserv for Family and Friends, we found that socioemotional topics dominated [22]. Thus, some of the socioemotional expression is potentially directed away from the Daily Digest.
  We also expected that people who are more recently diagnosed would be experiencing the most uncertainty about their situation and would therefore post more frequently in order to gain new information and support to help them make sense of their situation. Concurrently, we also expected that there would be people who are many years postdiagnosis who would post often to provide the benefits of their hard-won understanding to others.
2. Do the posts differ among people in different stages of the disease? If indeed diagnosis with a rare disease prompts a search for biomedical information, we expected that soon after diagnosis or in the early stages of the illness, people would be most likely to post *biomedical* questions, eg, about tests, symptoms, and medications, to help in managing their health, and that they would also be concerned with *organizational* issues such as finding a good treatment center and having the financial means or insurance coverage to pay for care. As time goes on and as the disease progresses, we expected that messages would be more concerned with seeking *socioemotional* support from others and reflecting on the impact of an increasingly disruptive illness on family relationships and emotional state.
3. To what extent do the messages on a support group's listserv reflect the psychosocial challenges and dilemmas identified in the social-scientific literature on the experience of chronic disease? The following themes are identified: uncertainty, role change and identity reconstruction, and stigma. We anticipated that these themes would be explored in many of the messages posted to the PBCers' Daily Digest.

## Method

Permission for the study was obtained from two sources: the Institutional Review Board of Lehigh University, Bethlehem, Pa, and the Board of Directors of the PBCers Organization.

Studies of computer-mediated support groups have generally used two approaches to understanding the role of such groups: content analysis of posts to listservs or websites, and surveys of users of Internet support websites to ask about their participation [12-14]. For the present study, we focus on 2 months of posts from the online Daily Digest, in March and September 2003.

### Coding System

Two different time periods, spring and fall of 2003, were selected to avoid possible seasonal bias. To analyze the posts, we created a codebook that expanded on Bales' [23,24] theoretical framework of human interactions that emphasized the dynamic tension between task and socioemotional activities in an environmental or organizational context. Bales' framework has also formed the basis for coding of online messages by other researchers [14,25].

For the PBCers Organization listserv, the primary *task* involves educating persons with primary biliary cirrhosis about the many aspect of the disease, its diagnosis and treatment. *Socioemotional* aspects are critical as well, especially for members providing peer support to one another. As Bales and others have articulated, the challenge is for an organization (or small group, or society) to maintain a functional equilibrium or balance between achieving its task goals and maintaining an acceptable level of cohesion. In this case, some members have met face-to-face, but for most, the interactions are via the listserv.

Bales' research showed that groups can become more formalized over time, develop norms for more or less emphasis on task or emotions, and achieve more or less problem solving. Following Bales' framework, we developed a coding schema to understand how this Web-based group met members' needs. We developed a coding system with three main categories—*biomedical* (corresponding to task), *socioemotional*, and *organizational/systems*.

We specified the *biomedical* category to include a set of subcategories designed to represent the range of biomedical topics that was relevant to this group, such as references to medications, symptoms, tests, and treatments as well as issues related to self-care and transplant, as well as the subcategory "*other biomedical*”.

The *socioemotional* category captured members interactions that had emotional content, such as fear and anxiety, hope, anger, frustration, the presence or absence of support from people in their lives, and support for others on the list. Included in the socioemotional categories are several codes designed to capture the psychosocial challenges of living with liver disease. These are “role change/identity”, “stigma”, and “thoughts about the future” (to capture expression of uncertainty about the future).

The *organizational* category was added as a special adaptation of the framework, recognizing that the listserv was also occasionally the place for discussions of broader topics, such as comments about the PBCers Organization, meetings, and fund-raising, or references to hospitals and financial issues.

We identified 12 topics in each category, based on our preliminary analysis of the Daily Digest during other months and informed by the coding systems employed by other researchers. Following Bales' Interaction Process Analysis coding system, we developed an equivalent number of subcategories in each category to facilitate analysis across categories.

Consistent with the Balesian approach, in each of these categories we included a code for ”seeking” either information or response, to distinguish those messages that involved asking about tests, insurance, etc from those that either reported information about the poster or provided information in response to questions.

### Coding Process

For this content analysis we used the act, defined as the simple sentence (or thought), as the coding unit. As with face-to-face conversation, there were multiple acts in each exchange or post. For the 2 months of posts, we quantified the online interactions according to the 36 topics and 3 categories. Each topic was operationally defined in a detailed codebook, enabling coders to attach specific labels to manifest content.

Each post was independently reviewed and coded by 2 coders, assigning relevant codes only once to each complete message (post), regardless of how many times a particular topic might be expressed in that message. The coding supervisor resolved questions, and the coders achieved over 95% interrater reliability.

We also recorded demographic information about the poster — gender, age, and time since diagnosis — when available. Gender was inferred from the poster's name unless it was ambiguous or from message content (eg, references to “my husband”). The information about age and date of diagnosis was most often included in the signature that many gave after all of their posts (a typical format for signature is name, age, state of residence, and year of diagnosis); thus it is unlikely that much more complete information on these variables could have been obtained from Daily Digests outside the time period under review.

### Validity

By using content analysis methods, we intrinsically had two key supports for validity. First, at the category level, we emphasized construct validity. We trained coders to understand the meaning of each (biomedical, socioemotional, and health systems). Further, we used only these 3 nonoverlapping categories so that no or little interpretation would be needed to determine the category. Second, at the topic (subcategory) level, in many instances the content itself provided face validity. For instance, a comment about a particular laboratory test is manifestly isomorphic with the item "tests/procedures." Coding for the topic was potentially more difficult, with 36 items. Coders were instructed, however, to determine the category first and then identify the topic. Thus, the task was quickly narrowed to selection from among only the 12 topics within the category.

In this analysis, the focus was on how individuals perceive their experience. We were not seeking concurrent validity, for example, with a physician's interpretation of the same data. Rather, our goal was to characterize the interactions in a way that was consistent with the intended meanings. Thus, if a person with primary biliary cirrhosis expressed worry when a laboratory report showed increased alkaline phosphatase, there would be socioemotional content even though that person's physician may view the same data as a normal fluctuation.

### Statistical Analysis

Data from the posts were analyzed in SPSS. Descriptive statistics were used; correlation (Pearson *r*) and tests of difference (*t* test) were applied to determine statistical significance of results.

## Results

### The People who Post

Table 1 presents available demographic data on the posters, as well as the mean number of messages posted. A total of 275 people posted 710 messages (posts) to the PBCers' Daily Digest in 2 months during the spring and fall of 2003. There was a range of 1 to 28 different messages per person, an average of 2.58 posts per person. The number of topics per post ranged from 1 to16 out of a total potential 36; the mean number of topics per post is 4.24.

Of the 250 people for whom information on sex was available, 239 (95.6%) were women and eleven (4.4%) were men. They ranged in age at the time of posting from 28 to 78 (mean= 54.8); almost three fifths (37 out of 63, 58.7%) were in their 40s or 50s, and most of the remainder were 60 or older. Almost a third (34 out of 105, 32.4%) had been diagnosed one year or less prior to their post, while almost one fifth (21 out of 105, 19.1%) had known about having primary biliary cirrhosis for 10 years or more (mean number of years since diagnosis is 5.1).

Of the 90 people who cited their stage of disease, exactly half were in the earlier (1-2) stages, and half in the later (3-4) stages. Of all those who posted, 22 people (8%) mentioned that they had had a transplant.

**Table 1:** Data on people who posted to Daily Digest, N=275

	N (posters)	Minimum	Maximum	Mean	Standard Deviation
Messages posted per poster	275	1	28	2.58	3.01
Age	63	28	78	54.8	11.1
Stage	90	1	4	2.6	1.1
Years since Diagnosis	105	0	26	5.1	5.1

### Emphasis of Posts

Consistent with our first hypothesis that a focus on biomedical information would dominate the listserv, topics in the biomedical category were almost twice as prevalent. Posts averaged 2.2 biomedical topics, 1.2 socioemotional topics, and 0.8 organizational/systems topics. See Table 2 for the proportion of posts that contain each of the 36 topics.

**Table 2:** The proportion of all posts that include the category/topic (N=710 posts)*

**Category/Topic**	**Nof Posts**	**% of Posts**
**Biomedical (Mean 2.2 topics per post)**	**531**	**74.6%**
Liver disease	57	8.0
Diagnosis/prognosis	126	17.7
Symptoms	183	25.7
Medications	220	30.9
Health care provider	233	32.7
Tests/procedures	184	25.8
Self-care behaviors	99	13.9
Other non-liver diseases	109	15.3
Transplant	63	8.8
Research	97	13.6
Other biomedical	36	5.1
Seeking biomedical information	142	19.9
**Socioemotional (mean 1.2 topics per post)**	**439**	**61.7%**
Spiritual/prayer	54	7.6
Negative emotions	136	19.1
Positive emotions	180	25.3
Thoughts about the future	28	3.9
Relationship to health care provider	49	6.9
Role change/identity	21	2.9
Stigma	5	0.7
Relationships with family and friends	44	6.2
Support to peers (e.g. others on the list)	289	40.6
Coping strategies	47	6.6
Other socioemotional	9	1.3
Seeking socioemotional response	14	2.0
**Organizational/Systems (mean 0.8 topics per post)**	**371**	**52.1%**
PBCers national organization (including Internet website)	125	17.6
PBCers/ALF fund-raising	57	8.0
Local PBCers activities	70	9.8
Hospitals/treatment organizations	62	8.7
Health care providers in general	46	6.5
Medical insurance	42	5.9
Social security/disability insurance	11	1.5
Pharmaceuticals	27	3.8
Financial issues	41	5.8
Employment issues	2	0.3
Other organizational/systems	33	4.6
Seeking organizational/systems response	64	9.0

^*^ For example, 8.0% of all posts included a comment about liver disease. Since the majority of posts included more than one topic, the percentages do not add up to 100%.

Of the 6 topics that appeared in 25% or more of all posts, 4 were biomedical: health care provider (32.7%), medications (30.9%), tests and procedures (25.8%), and symptoms (25.7%). Selections from posts that illustrate the most frequent biomedical topics follow. (Please note that to present meaningful examples, we gave quotes longer than the coding unit--the single thought, sometimes meaning that multiple topics are included.)

I went to my doctor's appointment this morning and after blood work was told that the reason I am jaundiced is because of the two units of blood I had transfused last Thursday. (*health care provider*)

My question is concerning the two different medications for PBC; my GI doctor insists that both medications are exactly the same and the only difference is in the company that manufactures the drugs. I talked to the pharmacy and was told that one of them is not on their formulary and that is why I cannot get a prescription for it. (*medications*)

We had an appointment this morning and he has agreed to run a battery of liver tests as well as an ultrasound of her liver and another possible liver biopsy.(*tests and procedures*)

Regarding your email about . . . nosebleeds, here is my one cent.. . . I have constant trouble with nosebleeds and they drive me bananas! . . . I don't know what to relate it to. (*symptoms*)

In light of the dominance of the biomedical category, it is noteworthy that the first and sixth most frequently used topics were in the socioemotional category. While the 2 major categories, *biomedical*and *socioemotional*, are analytically distinct, they are commonly joined together in the messages; biomedical topics are significantly correlated with socioemotional topics (*r*= .326, *P*<.001, n=710). The most frequent single topics were peer support (included in 40.6% of all posts) and positive emotions (25.3%).

Examples of these two topics follow:

Congratulations to you for taking that pre-transplant evaluation step. It is an enormous step--and you did it! (peer support)

Being a new member here at PBCers, it was a delight to hear from everyone . . . especially with all the wonderful information that was passed along to me. It is incredibly comforting to know that, even though our disease can be awful, we are all connected by this bond. (positive emotions)

The peer support example illustrates how support is often combined with a biomedical topic. Very often posts reflected a number of socioemotional topics together as well. The following post illustrates how these topics often appear in combination:

I am so thankful for all of you, for even though I have not participated on the chat line, silently you have all been helping me cope. I find not many of my wonderful family or friends can understand the roller coaster of emotions you face when dealing with a chronic illness.. . . I have two young children. . . . My only prayer is to live to see my grandchildren. . . . Here is what has helped me. . . . Positive thinking, fill your life with positive people, live like it is your last day.

### Differences in Frequency and Content of Posts by Age, Disease Stage, and Time Since Diagnosis

Posters who reported fewer years since diagnosis were more likely to be seeking biomedical information than those who are further in time from their diagnosis (*r*= -.241, *P*<.001, n=313). However, a poster's time since diagnosis is unrelated to seeking either socioemotional or organizational/systems responses, although it must be noted that the topic, “seeking socioemotional response,” occurs infrequently for the entire sample.

We also hypothesized that newly diagnosed people or those in the early stages of the disease would post more often, but the results of correlational analysis show no relationship between the number of messages posted and years since diagnosis or stage of disease. Only when stage is divided into early (1 and 2) and late (3 and 4) is there a nonsignificant trend towards a difference, but in the opposite direction from expected; those in later stages posted an average of 3.87 messages, compared to an average of 2.64 for people in earlier stages (*t*= 1.786, *P*=.08, n=90).

We also found no evidence for the expectation that there would be more messages from a group of people who had known about their primary biliary cirrhosis the longest time and were thus sharing the knowledge they had acquired. Table 3 shows that those who were more than 10 years since diagnosis did post more often, an average of 3.45 messages, compared to those with less than a year since diagnosis, who posted an average of 2.44 times. Yet the largest number was 3.59 messages from people who were 2 to 4 years post-diagnosis, and these differences are not statistically significant.

In contrast to our expectation that younger people would use the Internet more, within this sample, age was unrelated to either frequency of posts or to the content of the messages. There were not enough men to be able to make meaningful comparisons by gender.

**Table 3:** Year since diagnosis and number of messages posted

Years Since Diagnosis	N	Number of Messages
1 year or less	34	2.44
2-4 years	27	3.59
5-9 years	23	2.83
10 or more years	20	3.45
Total	104	3.02

### Psychosocial Themes.

Contrary to the expectations in our third hypothesis, the topics reflecting issues of role change/identity, stigma, and uncertainty (all identified in social-scientific literature as key concerns for people with chronic illness) appeared infrequently in this set of messages. “Role change/identity” appeared in 2.9% of messages. One example follows:

I am a grandmother and was the most active of women, a superwoman three years ago. Now I can hardly get out of bed sometimes.. . . I am mad most of the time, depressed, miss my family, feel guilty toward my husband. This PBC stinks.. . . I feel isolated.

References to “stigma” were almost non-existent (0.7% of messages). When comments about stigma did appear, they often focused on proposals to change the name of the disease in order to try to disassociate it from alcoholism. As one person wrote,

If a new name has not been “officially” adopted, can the patients vote to do it? It looks like the single thing we could all do to improve understanding and treatment of the disease, and our own “life chances”. . . Cirrhosis means only one thing to most people—alcohol abuse. Doctors label us as alcoholic, knowing nothing about PBC. How many people were asked how much they drank as soon as the AMA antibody showed up on their blood test?

“Thoughts about the future” appeared in only 3.9% of the posts. One example gives an indication that reading the Daily Digest can enhance as well as alleviate uncertainty about the future:

I have been reading some members' stories and am concerned about what's in store for me. I realize that it may be many years before I get to the final stages of this disease, but it may very well be sooner rather than later.. . . I am totally in the dark here and would feel a lot better if I knew more. Is there anyone else out there at the same stage of this disease, who shares the same concerns as I?

## Discussion

### Reasons for Using the Internet

We identified 3 major issues regarding the reasons that people with rare diseases use the Internet, in particular the PBCers' Daily Digest: balancing biomedical and socioemotional needs, validation via the Internet, and online group development.

### Balancing Biomedical and Socioemotional Needs

The primary finding is that the PBCers' Daily Digest has a biomedical, rather than socioemotional or organizational, emphasis. The Daily Digest acts as an informational resource, with participants sharing the empirical information they have gained from their own experiences and the research they have found. Additionally, the fact that individuals struggle with symptoms that are not understood or acknowledged by others as “real” motivates them to use the listserv as a resource for discussing their medical conditions with peers.

The PBCers Organization offers information in the context of support that is invaluable to individuals dealing with the emotional effects of primary biliary cirrhosis. Over 40% of the messages involve individuals giving or receiving peer support, indicating that people were likely to turn to a website that provides a supportive environment for obtaining biomedical information.

### Validation via the Internet

The PCBers Organization is valued in part because it serves a population with a rare disease. Personal posts testify that primary biliary cirrhosis is a disease not well understood by physicians and other medical professionals, and that the ability to correspond with others in the same situation is greatly appreciated. As expressed in one post, “Even though PBC is rare and doctors don't know much about PBC, my doctor is going to learn. We will learn together.” Both factors—the lack of medical validation and the rarity of the illness--may help to explain why a group that is mostly women does not follow the findings of Klemm et al [15] regarding women's emphasis on socioemotional communication in cancer lists but rather emphasizes the biomedical aspects.

As with some other autoimmune disorders affecting women, people with primary biliary cirrhosis experience significant fatigue, but it is hard for others to appreciate or understand because it is an invisible symptom. Studies of primary biliary cirrhosis, including our own, show that fatigue is not linked to age or years since diagnosis, and it is not appreciated by others as an objective or “real” symptom [26]. It is not surprising, therefore, that many people who have primary biliary cirrhosis feel that their experiences are not well understood or appreciated, and that the PBCers' Internetwebsite offers necessary validation as well as information and support. As one person wrote, not atypically,

I too believe these PBCers are angels. They have calmed my fears so many times. They have answered any questions I have asked. . . . They have done anything I have asked of them and they don't even know what I look like. Isn't that amazing? . . . It just astonished me that there are so many people who will take the time to do this service for us out here who aren't frightened about a disease even our doctors don't understand. I can't imagine life without our angels and I love each and every one of you.

### Online Group Development

The biomedical focus in the context of social support appears to reflect, in part, the organizers' priority. The leaders of the PBCers Organization have established alternative listservs for other topics, and posts from more ”senior” peer experts may provide role models for newer members. As with other self-help organizations, an emergent leadership may be important to the ongoing group culture. Research is needed to look explicitly at organizational leadership and emerging norms on the Internet.

### Differences Within the Primary Biliary Cirrhosis Population in Posting to the Daily Digest

More recently diagnosed people post more messages seeking biomedical information, as predicted. The Daily Digest gives people an opportunity to find out more about ambiguous symptoms, the relative merits of different medications and their possible side effects, as well as the meaning of different diagnostic tests. For example, many times new members have questions about what they might expect from a liver biopsy that has been recommended.

Newly diagnosed people, contrary to our hypothesis, do not post more messages overall. At different points in the illness, people with primary biliary cirrhosis have different concerns to communicate. Those who are more experienced with the disease do often provide answers and encouragement in response to the posts of others, but they do not dominate the discussion. There were no differences by age in the frequency of posting.

### Psychosocial Challenges and the Internet

We found few mentions of the key issues raised in the literature on chronic disease—uncertainty, role and identity change, and stigma. There are several possible explanations: these are not really salient issues to the people who post to the Daily Digest; our coding system is not sufficiently sensitive to capture these themes; or the Daily Digest, with its emphasis on exchanging biomedical information and encouragement, is not the forum for discussion of these problems. Supporting the last possibility, in-depth interviews have given us some insight into the importance of these challenges in the lives of people with primary biliary cirrhosis (EDS and JNL, unpublished data, 2005).

With regard to stigma specifically, it is possible that this is not much of an issue in the case of primary biliary cirrhosis because the major symptoms (fatigue and itching) are invisible. Yet some studies indicate that people with nonvisible symptoms do fear being stigmatized for complaining about their condition or not being able to fulfill their social roles [27,28]. As Wainwright [19] and others have found, the association of liver disease with substance abuse is problematic for many. Our current research looks more closely at the role of stigma with primary biliary cirrhosis.

### The Use of the Internet for Health-Related Purposes

Many concerns have been raised about disadvantages of relying on the Internet for information and for support. For example, more than 79 studies have evaluated the accuracy, completeness, and comprehensibility of health-related websites, mostly coming to negative conclusion [29]. Han and Belcher's survey of parents using Internet support groups [12] revealed dissatisfaction with the lack of physical contact, the large volume of mail, including its use for unrelated topics, and the impact of receiving bad news about children who died. In contrast, Potts and Wyatt's [30] study of doctors' experiences of Internet-using patients found that despite concerns about misinformation, the doctors still praised the benefits of information, advice, and social support.

Online groups are not only easier to access for people who are geographically remote from face-to-face support groups, but they also have the potential to involve those who might not attend a group even if it were available nearby. Klemm and Hardie [31] discovered this when they compared cancer patients participating in online support to those in a face-to-face group; the online participants were significantly more depressed than those in the face-to-face group, suggesting that the Internet may provide an important outlet for people who might otherwise not attend the more traditional type of support group. It is noteworthy that 14.8% of the people we surveyed at the national PBCers Organization conference (EDS and JNL, unpublished data, 2005) reported being in stage 4 of the disease, the most advanced, while twice as many (28.9%) of people posting to the Daily Digest, who gave their disease stage, are in stage 4. As was found with depression, one might conclude that people with severe disease are more likely to connect with others online rather than in person.

### Limitations of the Study

People who use the Internet for health and other purposes have been found to be younger, and more educated and affluent than those who do not [9,32]. A possible limitation of this study is that people who read and post to the PBCers' Daily Digest are more educated than the general population of people with primary biliary cirrhosis, a population for whom demographic characteristics are not known. Yet it is also likely that they are not younger on average than all people with primary biliary cirrhosis, who tend to be mostly in their 40s and 50s.

A further limitation of the Daily Digest data is that information on age, stage of disease, and time since diagnosis is not available for many of those who posted. Thus conclusions about differences in messages related to these factors must be considered with caution. It is also the case that we only have information from those who post, and studies of “lurkers” suggest that the majority of people who connect to online message boards do not post for a variety of reasons [33, 34].

On the other hand, posters represent a much larger group of people with primary biliary cirrhosis who are located all over the United States and in several other countries. Data from our survey (EDS and JNL, unpublished data, 2005) show that even among those who are sufficiently connected to the PBCers Organization to attend a national conference, only about one fourth (25.7 %) post to the Daily Digest on any regular basis, while more than 3 times as many (82.3 %) read it very regularly. There were no demographic differences between those who posted regularly and those who did not (ie, the lurkers on this list).

Table 1 indicates a wide range of messages posted per person, from 1 to 28. Over 99% of the people in this sample posted less than 20 messages. To see if the outliers (2 people who posted 20 or more messages) influenced the overall results, we eliminated all of their messages and redid all the analyses, with no significant change in results. Findings in Table 2 for individual topics changed less than 1%, except for the total socioemotional category, which rose from 61.7% of total messages to 63.1%, and the total organizational/systems category, which increased from 52.1% to 54.1% of all messages.

In conclusion, these data suggest that the Internet provides a highly valued outlet for people who have a rare disease, primary biliary cirrhosis. It appears to be particularly valuable for those who are newly diagnosed and in need of health information, but it is an important resource for people at all stages of the disease. The focus on biomedical issues, often framed in the context of offering support to others, makes this Internet-based organization an important tool in helping people with chronic illness address the problems raised by Charmaz [18] of making sense of bewildering symptoms, reconstructing order, and maintaining control over life. People with primary biliary cirrhosis help each other through the Daily Digest to understand the disease process and its impact on their lives in an environment of encouragement and reassurance.

Research that compares those with primary biliary cirrhosis who are involved in an Internet support group and those who are not would be an important next step to better understanding the role of the Internet in patients with chronic liver disease and the implications of it on the course of the disease.
